# Editorial: Biosynthesis, regulation, and ecological roles of plant volatile organic compounds

**DOI:** 10.3389/fpls.2026.2018095

**Published:** 2026-10-19

**Authors:** Tuo Zeng, Jianyong An, Caiyun Wang, Hao Hu

**Affiliations:** 1 Guizhou Key Laboratory of Forest Cultivation in Plateau Mountain, College of Life Sciences, Guizhou Normal University, Guiyang, China; 2 King Abdullah University of Science and Technology, Thuwal, Saudi Arabia; 3 National Key Laboratory for Germplasm Innovation & Utilization of Horticultural Crops, College of Horticulture & Forestry Sciences, Huazhong Agricultural University, Wuhan, China; 4 Center for Bioinformatics, School of Life Sciences, Faculty of Science, Peking University, Beijing, China

**Keywords:** chemical ecology, plant defense, plant volatile organic compounds, plant–environment interactions, pollination ecology, volatile biosynthesis, volatile signaling

## Introduction

Plants synthesize and release a wide range of volatile organic compounds (VOCs). These compounds are important components of floral and fruit aromas and also act as chemical signals that mediate interactions between plants and their biotic and abiotic environments. Plant VOCs are chemically diverse and include terpenoids, volatile phenylpropanoids/benzenoids, fatty acid derivatives, and various sulfur- and nitrogen-containing compounds. Their biosynthesis and emission are regulated by genetic background, developmental stage, tissue type, and environmental conditions.

Plant VOC research has increasingly moved from descriptive profiling toward understanding the mechanisms that control VOC formation and ecological function. Advances in genomics, transcriptomics, and metabolomics have linked VOC variation with developmental programs, genetic variation, and environmental cues, while physiological and behavioral studies have clarified their roles in defense, plant–plant communication, and pollinator attraction. Recent work has also begun to reveal how receiver plants perceive airborne VOCs through processes including Ca^2+^-dependent signaling and *KAI2*-mediated perception (Aratani et al., 2023; Stirling et al., 2024). Against this background, the nine articles in this Research Topic address VOC biosynthesis and developmental regulation, environmental and cultivation-dependent variation, postharvest effects, and ecological interactions.

## Biosynthesis and developmental regulation of plant VOCs

Plant VOC profiles often change markedly during development. In *Hesperis oreophila*, the volatile profile shifted markedly as flowers progressed from the bud stage to initial and full bloom, with terpenoids becoming the dominant floral volatiles at later developmental stages. These changes were accompanied by clear developmental stage-specific expression of genes involved in the MVA and MEP pathways and of multiple *terpene synthase* (*TPS*) genes. Candidate genes including *MVK*, *DXS*, and *TPS22* were closely associated with the accumulation of characteristic terpenoids Xu et al. These findings indicate that floral scent formation is developmentally regulated and is associated with coordinated changes in terpene biosynthesis during flower development.

Terpene metabolism is also highly diverse. Xu et al. reviewed nearly 300 terpenes reported in tobacco and summarized their biosynthesis, key genes, and ecological functions. These terpenes contribute to tobacco aroma and also participate in defense against herbivores and pathogens. Taken together, these studies support the view that plant VOC phenotypes are shaped by precursor availability, key biosynthetic enzymes, and transcriptional regulation, rather than simply reflecting changes in the abundance of individual metabolic end products.

## Environmental and postharvest modulation of plant VOCs

Plant VOCs show considerable environmental plasticity. Even within the same species or genotype, VOC profiles can vary substantially with developmental stage, habitat, cultivation practice, and postharvest processing. Yang et al. compared the floral VOC profiles of *Panax notoginseng* grown in agroforestry systems with *Pinus armandii* or *P. yunnanensis*. Terpenes were the predominant VOCs, whereas their composition and relative abundance differed markedly between the two cultivation systems. Further analysis identified humidity as the major environmental factor associated with VOC variation, followed by temperature. Peng et al. showed that regional climatic and soil conditions were associated with changes in lipid and terpenoid metabolism, including the accumulation of specific volatiles such as (−)-camphene and (Z)-carveol.

Postharvest treatment and analytical methods can further influence the VOC profiles that are detected. Guo showed that flower condition and extraction method substantially affected volatile profiles of white champaca, with methyl anthranilate and linalool contributing strongly to the characteristic aroma of different samples. Sun et al. further combined volatile metabolomics, relative odor activity values, and sensory evaluation to assess the effects of cultivar background and processing method on the fruity–floral aroma of black tea. Their results showed that genotype and processing jointly shaped the final volatile composition and aroma characteristics.

Together, these studies show that plant VOC profiles are dynamic traits shaped by both genetic and environmental factors, as well as by cultivation and postharvest processing. Studies have similarly shown that soil conditions, pollination, herbivory, and their interactions can drive rapid divergence in floral scent traits across generations (Dorey and Schiestl, 2024). Future studies should therefore consider not only the genetic regulation of VOC biosynthesis but also environmental context and methodological variation.

## Ecological functions of plant VOCs

Plant VOCs can trigger physiological and behavioral responses in other organisms and thereby mediate both antagonistic and mutualistic interactions. Pyrethrum illustrates this functional diversity. (E)-β-farnesene mimics the aphid alarm pheromone, reducing aphid colonization while attracting aphid predators, whereas nocturnal emission of (−)-germacrene D from open disk florets attracts moth visitors and promotes pollination success (Li et al., 2024; Zeng et al., 2024). These findings illustrate how different VOCs can mediate contrasting ecological functions within the same plant species.

Plant VOCs also participate in plant–plant communication. Using non-invasive real-time imaging. Kinoshita et al. showed that communication can occur even in an *Arabidopsis* accession unable to produce typical green leaf volatiles. Cytosolic Ca^2+^ signals were detected in neighboring undamaged plants, while acetophenone and alkanes accumulated after insect damage, suggesting that these compounds may contribute to GLV-independent communication. The behavioral effects of plant volatiles are not restricted to insects. Wang et al. showed that volatile and flavor-related metabolites in wheat influenced bird feeding preferences, with n-decanol associated with preference and 2-methyl-1-butanethiol with avoidance. Aphid infestation also induces antioxidant enzymes and phenylpropanoid-related defense responses, with substantial variation among wheat genotypes Kumari et al. These findings emphasize that VOC-mediated defense operates as one component of a broader inducible defense network involving multiple biochemical pathways.

## Encoding and decoding plant volatile signals

Collectively, the studies in this Research Topic point to a shift from cataloguing VOCs toward understanding how volatile signals are generated, released, perceived, and translated into ecological outcomes. Many studies still rely mainly on correlations between VOC abundance and gene expression, environmental variables, or behavioral responses, whereas direct causal links remain limited. The molecular basis of VOC perception is particularly poorly understood, as only a small number of receptors and receptor-like proteins have been characterized despite the structural diversity of VOCs perceived by plants (Bergman et al., 2026).

Future research should therefore address not only which VOCs are released, but also when and where they are produced, how information is encoded in the composition and timing of volatile emissions, how receiver organisms decode these signals, and how these interactions affect plant survival and reproduction. Establishing such links across molecular, organismal, and ecological scales will be important for translating VOC biology into applications in pest management, pollination, crop quality improvement, and sustainable crop production.
